# *Eichhornia crassipes*: a Powerful Bio-indicator for Water Pollution by Emerging Pollutants

**DOI:** 10.1038/s41598-019-43769-4

**Published:** 2019-05-13

**Authors:** Chloe De Laet, Théodora Matringe, Eddy Petit, Claude Grison

**Affiliations:** 1 Laboratory of Bio-inspired Chemistry and Ecological Innovations, UMR 5021 CNRS - University of Montpellier, Montpellier, France; 2 IEM, CNRS - University of Montpellier - ENSCM, Montpellier, France

**Keywords:** Invasive species, Pollution remediation

## Abstract

*Eichhornia crassipes* is well known as an invasive aquatic plant. It is also used very effectively in phytoremediation, particularly for the rhizofiltration of effluents contaminated by heavy metals. In this article, we show that it is also an excellent bioindicator of water polluted by worrying organic pollutants such as endocrine disruptors and neonicotinoids. As a proof of concept, di-*n*-hexylphthalate, pentabromodiphenyl ether, nitenpyram, acetamiprid and bis (3-*tert*-butyl-4-hydroxy-6-methylphenyl) sulfide were clearly identified by UHPLC-HRMS or GC-MS in the root system of *E. crassipes* after a short period of exposure. These results open up new perspectives for the remediation of water polluted by alarming organic pollutants.

## Introduction

*Eichhornia crassipes*, commonly known as water hyacinth, is one of the aquatic plants that has attracted the most scientific interest in the last decade. Originally from South America, its ornamental appeal led to its introduction into Africa, Asia, the South Pacific, North America and Europe, where it has become invasive^1,2^. Its great capacity to multiply has become a real problem in the tropics, where high temperatures and a lack of predators has led to its uncontrolled development. In Europe, it has recently been included in a list of invasive alien species (Implementing Regulation No 2016/1141 Commission 13 July 2016 JOUE No L 189, 14 July). One hundred and nine scientific articles describe the problem.

However, *Eichhornia crassipes* is not just an invasive and harmful plant, it is also a useful plant with remarkable metal pollutant phytoaccumulation capicities. *E. crassipes* is capable of bioconcentrating toxic metals such as Cr, Cu, Co, Ni, Zn, Pb, Cd, and As in its root system. Its performances have been described in more than 620 articles since 2008^3–7^. In addition, our group studied the adsorption efficiency and adsorption capacity of each pre-cited metal, and highlighted their remarkable performances compared to other aquatic plant species^8^. Moreover, *E. crassipes* is also known to multiply easily and to form an amazing quantity of root biomass^9^. Thanks to the biological characteristics and morphology of *E. crassipes* our group for implemented large-scale effluent rhizofiltration. In addition to its ability to remove pollution from industrial water, *E. crassipes* is also capable of extracting strategic metals such as Pd by rhizofiltration, providing an effective and simple method for recycling precious and expensive metals. Since no translocation was observed during metal uptake, roots were separated from the rest of the plant in order to recycle metals^8^. Our group has taken part of this natural advantage by transforming the metal-rich root system into ecocatalysts, useful tools for organic synthesis. The concept of recycling by rhizofiltration/valorization by ecocatalysis was developed with the trace elements Pd, Pt, Rh and Cu^8–14^.

Some authors have recently described the use of *E. crassipes* for the phytodegradation of emerging pollutants. Kang and Kondo^15^ described the possible biodegradation of bisphenol A (2,2-bis (4-hydroxyphenyl) propane) by water hyacinth, while Xia and Ma^16^ described the degradation of ethion (O, O, O′, O′-tetraethyl S, α-methylene diphosphorodithioate). The ethion initially present in water, was found in hyacinth plants in shoots (54 to 91%) and in roots (73 to 81%). But the ethion concentration decreased after 1 week of incubation in shoots and in roots. The authors concluded that the decrease of ethion in water hyacinth might be caused by phytodegradation.

Ismail *et al*.^16^ demonstrated the capacity of *E. crassipes* for the uptake of non-metallic inorganic compounds: nitrate, orthophosphate, nitrite and ammonium, with a better performance for nitrate. These observations were confirmed by Valipour *et al*.^17^ who also showed that the roots can extract nutrients (nitrates and phosphates) which are translocated and stored into shoots.

Far fewer studies have focused on investigating the physicochemical properties of *E. crassipes*’ root system to explain phytoremediation performances of the plant. DellaGreca *et al*.^18^ were among the first to describe the existence of new “phenylphenalene” structures extracted from water hyacinths. They identified two interesting compounds, phenyl naphthalenedicarboxylic acid and a dimeric oxidized phenylphenalene skeleton, that might play a role as phytoalexins and phytoanticipins. Later on, Luo *et al*.^19^ found new phenylphenalene compounds: hydroxyanigorufones. The discovery of these structures was an important finding as they could be involved in allelopathic interactions impeding the development of other organisms through eluviation (leaching), root exudation or volatilization. However, these phenylphenalene compounds account for only a minute fraction of the root structure (<0.1%) and therefore cannot explain the high affinity of *E. crasssipes* for metals.

Following their research on allelopathic effects, Shanab *et al*.^19^ published a paper on the presence of phthalate derivatives (ethylhexyl, methyl-dioctyl, di-isooctyl) in *E. crassipes* extracts. The authors showed the antimicrobial and antialgal activities of these aromatic compounds.

More recently, Carduso *et al*.^20^ found a high concentration of shikimic acid in *E. crassipes*. This acid is an important intermediate in the biosynthesis of aromatic compounds in plants and microorganisms (shikimic pathway). However, the carbon skeleton of phthalates does not derive from the aromatic skeleton of the shikimic pathway; phthalates cannot be biosynthesized from any known biosynthetic pathways in plants. In this article, we hypothesize that *E. crassipes* might be capable of phytoaccumulating phthalates among various organic pollutants. We tested the accumulation abilities of first living water hyacinth by rhizofiltration, and secondly dried roots in powder form by biosorption, in solutions enriched with two families of emerging organic pollutants: endocrine disruptors and neonicotinoids.

## Results and Discussion

**Figure 1:**
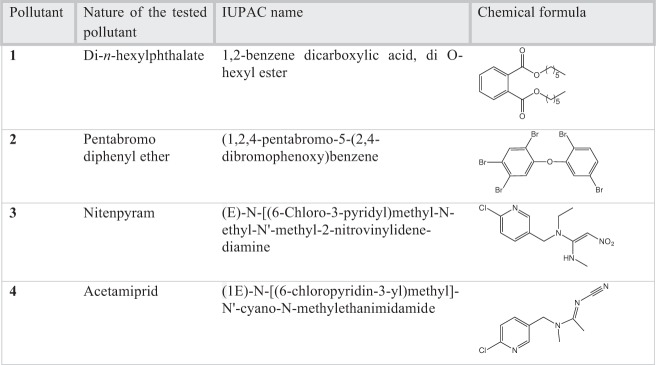
Nature of the tested pollutants.

The bioaccumulation capacities of organic pollutants by *E. crassipes* was first investigated. To do so we selected 4 different types of organic pollutants to be tested: di-*n*-hexyl phthalate **1**, pentabromodiphenyl ether **2**, nitenpyram **3** and acetamiprid **4**. Di-*n*-hexyl phthalate **1** is a monomer required for the production of plastics and pentabromodiphenyl ether **2** is a flame retardant; both are endocrine disruptors. Nitenpyram **3** and acetamiprid **4** are neonicotinoids, i.e. neurotoxic insecticides. All the pollutants studied are listed in Fig. 1. From a structural point of view, **1**, **2**, **3** and **4** are compounds which exhibit aromatic cycles. They present also some similarities with the phthalates identified by Shanab *et al*.^19^ in *E. crassipes*.

The contaminated solutions were prepared by dissolving each organic pollutant in water or in a mixture of water/isopropanol for the compounds insoluble in aqueous medium (compounds **1** and **2**). The accumulation abilities of *E. crassipes* were investigated in two parts:

- an entire living plant of *E. crassipes* was placed in the contaminated solution. Shoots and roots were harvested separately and were treated with methanol. The organic compounds were extracted in the methanol layer and were analyzed by UHPLC-HRMS (for compounds **3** and **4**) and GC-MS (for compounds **1** and **2**).
- dried roots in powder form were introduced in the contaminated solution. After stirring, the powder was separated by filtration and treated with methanol. The organic compounds were extracted in the methanol layer and were analysed by GC-MS.

The methanol layers were compared with the commercial references diluted in methanol (compounds **1** to **4**). As a control sample, water hyacinth plants were not subjected to any contaminated solutions but simply grew in water and were analysed as described previously by GC-MS.

### Study of the control plants – not subjected to contamination

#### Analysis of crude root extracts

**Figure 2:**
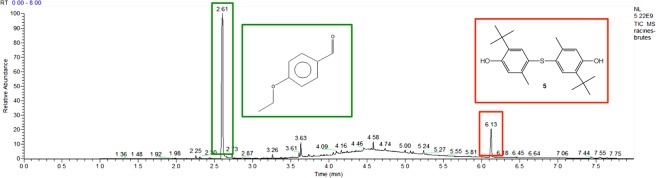
GC-MS spectrum of control root extracts.

No organic compounds were detected in the methanol layer of extracted shoots. However, the composition of root extracts was very intriguing and interesting. Two compounds were clearly identified after comparison with commercial samples: 4-methoxy benzaldehyde (2.61 min of retention time) and bis (3-*tert*-butyl-4-hydroxy-6-methylphenyl) sulfide **5** (6.13 min of retention time) (Fig. 2).

It was not surprising to find 4-Methoxy benzaldehyde in the plant root extracts. The biosynthesis of 4-Methoxy benzaldehyde is derived from the degradation products of lignin catalysed by the H_2_O_2_-activated peroxidase^21^. 4-Methoxy benzaldehyde probably corresponds to a degradation product of lignin, which is abundant in roots.

**Figure 3:**
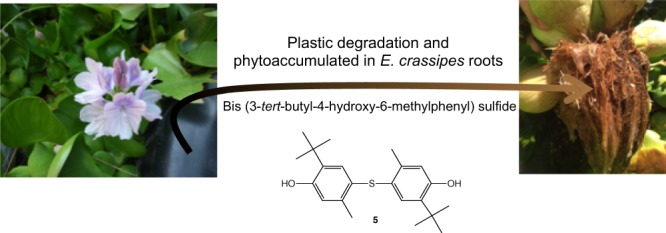
Biosorption of thioether 5 from tarpaulin.

The presence of thioether **5** was more surprising. Its structure corresponds to santanox or santox, which is an antioxidant used for the manufacture of tarpaulin used to line artificial ponds. Its presence in roots rather unexpected was then investigated. A few milligrams of tarpaulin consisting of vulcanizable EPDM rubber mixtures were cut and dissolved in isopropanol at 40 °C for 24 hours and analysed by GC-MS (Fig. 3). A peak having a similar retention time (6.13 min) could be attributable to santanox from the root extracts. Thioether **5**, used for the manufacture of the tarpaulin lining the water ponds used to grow water hyacinths, was thus found in their roots. This control experiment reflects the capability of *E. crassipes* to extract pollutants derived from aromatic structures.

#### Analysis of root extract fractions after chromatography

**Figure 4:**
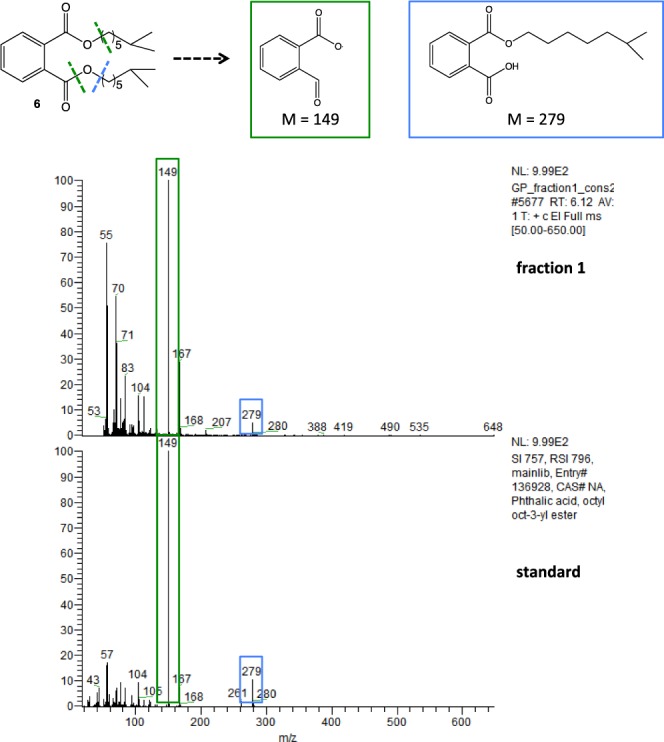
Identification of diisooctyl phthalate **6** by GC-MS.

In addition to 4-Methoxy benzaldehyde and thioether **5**, the GC-MS spectrum of the crude root extracts revealed a complex mixture of compounds between 3.5 and 5.5 min of retention time. The root extracts were fractionned using flash chromatography to isolate possible organic pollutants from the complex mixture. The GC-MS analysis of the non-vegetal compound, isolated from fraction 1, showed a retention time and a mass spectrum identical to diisooctyl phthalate **6**. The mass spectrum presented the fragments at 279 and 149 characteristic of phthalate structure (ions mono isooctyl phthalate and C_8_H_4_O_3_ respectively) (Fig. 4). No non-vegetal compounds could be clearly isolated from the other fractions of the chromatography.

Due to the unexpected presence of diisooctyl phthalate **6**, these analyses were repeated one year later (June 2018) and yielded identical results; the presence of **6** was once again detected. This result can be related to the study of Shanab *et al*.^19^ who identified as well 4 phthalates in the plant extracts. However the water hyacinth plants grow in opposite conditions: Shanab *et al*. collected wild plants in River Nile (El-Zomor canal, Egypt) while we used plants that were cultivated in greenhouses under controlled conditions (Occitanie, France). Two explanations can be advanced: Shanab *et al*. assumed that phthalates could derive from secondary metabolites from *E. crassipes*. However, phthalates cannot be biosynthesized from any known biosynthetic pathways in plants. Therefore we assume that *E. crassipes* exhibits an unusual capacity of phytoaccumulating phthalates, that derive from plastic and are widely spread in environment.

### Study of the root extracts – subjected to contamination

#### Analysis of phytoaccumulation capabilities of living *E. crassipes* by rhizofiltration

In the order to confirm the hypothesis that *E. crassipes* phytoaccumulates organic pollutants, *E. crassipes* plants were placed in contaminated solutions with the 6 emerging pollutants: di-*n*-hexyl phthalate **1**, pentabromodiphenyl ether **2**, nitenpyram **3** and acetamiprid **4**. After treatment, previously described, the methanolic root extracts were analyzed by UHPLC-HRMS (and GC-MS for compounds **1** and **2**).

**Figure 5:**
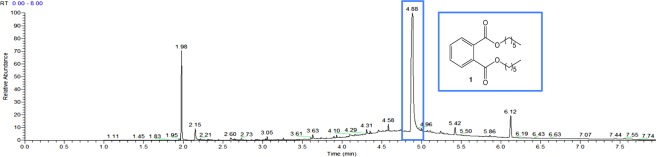
GC-MS spectrum of root extracts enriched in phthalate **1**.

Di-*n*-hexyl phthalate **1** was clearly observed by GC-MS at 4.88 min of retention time in the methanolic root extracts (Fig. 5). The characteristic fragment of phthalate structure (ion C_8_H_4_O_3_^4^) at 148.84 was observed. A co-injection of the extract and commercial di-*n*-hexyl phthalate was consistent with the interpretation. Compared to the control extract (Fig. 1), the presence of phthalate **1** confirms the capacity of *E. crassipes* to phytoaccumulate phthalate, which is in agreement with our initial hypothesis.

**Figure 6:**
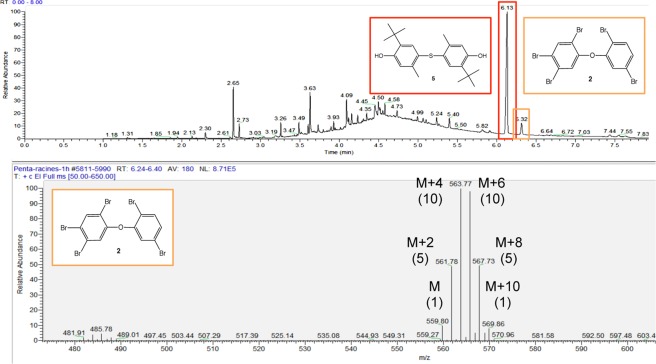
GC-MS spectrum of root extracts enriched in pentabromodiphenyl ether **2**.

Pentabromodiphenyl ether **2** was clearly observed by GC-MS at 6.32 min of retention time in the methanolic root extracts (Fig. 6). The mass spectrum is consistent with the typical isotope distribution of five bromine atoms calculated with isotope software M, M + 2, M + 4, M + 6, M + 8, M + 10, and the pattern 1: 5: 10: 10: 5: 1. Similarly to compound **1**, *E. crassipes* was able to bioconcentrate pentabromodiphenyl ether **2**.

Structurally, **2** is close to **5** (6.13 min), since **2** is a diphenyl ether and **5** is a thiodiphenyl ether. The phytoaccumulation of **2** is not surprising since **5** is systematically present as shown in the control sample (Fig. 2).

The phytoaccumulation studies of **3** and **4** neonicotinoid derivatives could not be performed by GC-MS due to their chemical properties. The extraction of nitenpyram **3** by *E. crassipes* roots was studied by UHPLC-HRMS, a high performance mass spectrometer. This machine is a Q-TOF (Quadripoliare-Time Of Flight) with a High-Resolution (DeltaM/M > 48 000) and a high accuracy (<3 mDa) and allows us to determine the Elemental Composition of a Mass (see Methods).

The identification of the nitenpyram **3** in the root extracts was achieved by comparing HRMS in mode TOF MS ES^+^ with the standard compound. 5446 structures were evaluated with 48 results within limits, and only one compound with a 99.94 percent degree of confidence could be attributed to **3** (see Methods Fig. 10).

The identification of the acetamiprid **4** in the root extracts was achieved by comparing HRMS in mode TOF MS ES^+^ with the standard compound. 372 structures were evaluated and only one compound with a 100.00 percent degree of confidence could be attributed to **4** (see Methods section). The mass spectra is consistent with the typical isotope distribution of chlorine, its pattern 6: 1 and the parity of mass (M + 1 = 223.0750 in ESI positive) due to the four nitrogen atoms.

#### Comparison with the control not subjected to contamination

The rhizofiltration studies on the six pollutants found in the root extracts of *E. crassipes* allowed us to develop specific and accurate analytical tools for their detection in non-enriched environment. We revisited the composition of the root extracts of our plant control, *E. crassipes* that was not subjected to any contaminated solutions.

Surprisingly all pollutants (**1**, **2**, **3** and **4**) were identified in the control root extracts after fractionation by liquid chromatography. If the phytoaccumulation of the phthalate **1** and thioether **5** in the control samples has been explained, the presence of neonicotinoids in *E. crassipes* not subjected to a solution enriched in neonicotinoids was unexpected.

Investigations near the production site revealed that neonicotinoids have been used for non-aquatic crops located close to the ponds. This observation can explain the presence of **3** and **4** in untreated roots. This demonstrates how *E. crassipes* is able to concentrate neonicotinoids and behaves as a powerful bioindicator. However, there was no direct local explanation for the presence of compound **2**, the flame retardant. It was probably due to an indirect contamination of the aquatic plant culture.

#### Analysis of phytoaccumulation capabilities of dried roots of *E. crassipes* by biosorption

**Figure 7:**
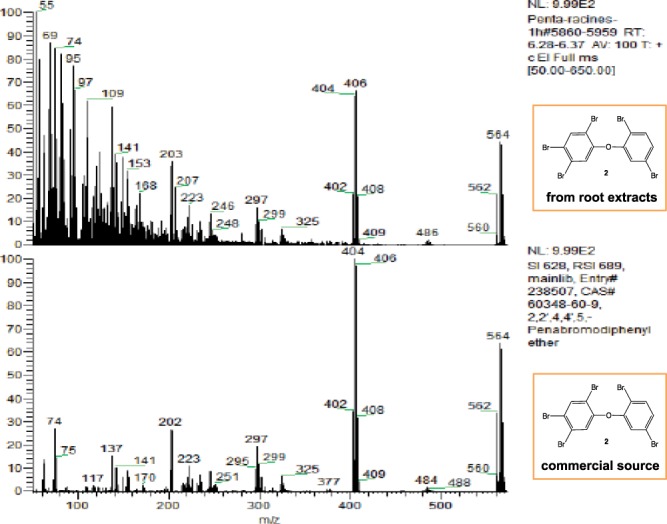
Comparison of isotopic signature of pentabromodiphenyl ether **2** between extract and commercial sample.

Encouraged by all these results, we decided to study the biosorption ability of *E. crassipes* roots in dehydrated powder form. Dihexyl phthalate **1** and pentabromodiphenyl ether **2** were selected as model pollutants for the study. Dried roots in powder form were introduced in the contaminated solutions of **1** and **2**. After stirring, the powder was separated by filtration and treated with methanol. The organic compounds were extracted in the methanol layer and were analyzed by GC-MS. Both dihexyl phthalate **1** (4.88 min) and pentabromodiphenyl ether **2** (6.32 min) were clearly identified by GC-MS: **1** was identified by its characteristic fragment at 148.84 (ion C_8_H_4_O_3_) in MS spectrum and **2** by its isotopic signature characteristic of pentabrominated products presenting mono and dibrominated fragments in MS spectrum (Fig. 7).

**Figure 8:**
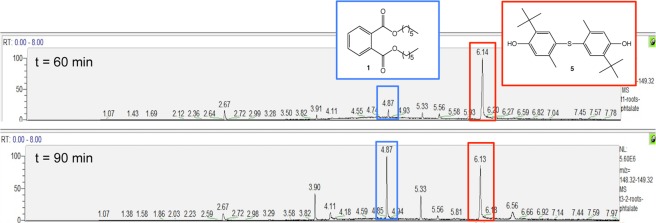
GC-MS spectra of root powder treated by dihexyl phthalate **1** (SIM mode).

The kinetic monitoring of dihexyl phthalate **1** presence (4.87 min) was demonstrative of the rapid biosorption by the dried roots. The follow-up was performed by SIM (Selected ion monitoring) scanning mode such that only the characteristic fragment of phthalate structure (ion C_8_H_4_O_3_) at 148.84 was detected (Fig. 8).

Considering the capacities of root powder to biosorb phthalates, an accurate quantification of biosorption was carried out. The quantity of phthalate extracted was assessed by subtracting the residual amount in the solution from the initial amount. Due to the hydrophobic nature of phthalates, the adsorption of phthalates on the walls of the reactor was controlled. No phthalate adsorption on the walls of the reactor could be detected, which demonstrated that the decrease of phthalates in solution was attributable to the biosorption by root powder.

The factor of biosorption, the measurement range, accuracy, relative standard deviation RSD were considered to quantify the biosorption (See experimental section). After validation of the calibration using commercial solutions and dodecane as the internal standard, it was possible to establish that 28% of the phthalates (mean of four replicates) were extracted from the concentrated solution (10 mg/L), which corresponded to 0.7 mg of phthalates per 39 mg of dried roots.

### Discussion on the mechanism of biosorption by *E. crassipes*

We showed that living *E. crassipes* is able to phytoaccumulate the pollutants **1**, **2**, **3** and **4** in natural conditions and that *E. crassipes* roots in dehydrated powder form presented biosorption capacities for **1** and **2**. Compounds **1**, **2**, **3** and **4** share a common structural feature, aromatic cycles. Considering the lipophilic character of the aromatic structure, we hypothesized an affinity of the organic pollutants for phenylphenalen structures to phospholipids naturally present in the plant.

We carried out a total lipid extraction of roots, following the procedure described by Folch *et al*.^22^, to examine the role of lipophilic structures in the biosorption procedure. A new biosorption of di-n-hexylphthalate **1** of roots lacking phospholipids was performed, but the results were not modified.

**Figure 9:**
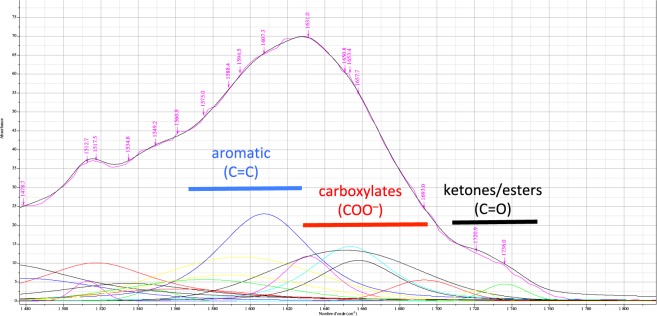
ATR-FT-IR spectrum of *E. crassipes* roots powder.

The organic pollutants must have an affinity to another component of water hyacinth roots. FT-IR is an ideal procedure to analyze biomass, entirely exploring the structural information of samples. ATR-FT-IR was used to analyze the *E. crassipes* roots powder components (Fig. 9).

The presence of aromatic compounds and carboxylic acids was unambiguously confirmed by Infrared Spectroscopy (IR) analysis using Fourier Transform Infrared Spectrometry (FT-IR).

After a mathematical fitting, (see Supporting information), we could observe aromatic compounds, which show absorptions in the region around 1607, 1594 cm^−1^ due to carbon-carbon stretching vibrations in the aromatic ring. In turn, carboxylate units are characterized by four intense bands at 1657, 1653, 1650 and 1631 cm^−1^ corresponding the carbonyl stretch C=O from the COO^−^ group. We determined the area ratio of the C=O bond vibration band and the aromatic ring vibration bands.

The fitting of the ATR-FT-IR spectrum clearly shows that root powder from *E. crassipes* is rich in carboxylate functions. This material is suitable for the biosorption of metallic elements based on the formation of metal carboxylate salts^8^. The metal salts can interact with the Lewis base sites of the pollutants, which are di-carboxylic ester, 1,2-dibromophenyl, pyridine-vinylidenediamine, pyridine-acetamidine, phenol for **1**, **2**, **3**, **4** and **5** respectively, and can participate to its biosorption.

The fitting of the ATR-FT-IR spectrum also shows the presence of aromatic compounds that can derive from lignin, a well-known component of roots. The aromatic structure of lignin possesses a lipophilic part, which could interact and retain the aromatic pollutants.

We can anticipate that the association of lignin and metals in roots favour the biosorption of aromatic organic pollutants.

## Conclusion

In this article, we showed for the first time that *E. crassipes* can bioconcentrate emerging organic pollutants such as endocrine disruptors and neonicotinoids. The aquatic plant can be used as a bioindicator of pollution, but also as a natural remediation tool provided it is introduced in a controlled environment and in powder form. Using the root system in powder form presents three interesting solutions to important environmental problems:

- it can prevent the dispersion of emerging organic pollutants into the environment.
- using dead plants in powder form instead of living plants prevents the development of complex installation for rhizofiltration. The process of biosorption is easy and quick compatible with scale-up. The use of invasive plants such as *E. crassipes* does not pose an environmental threat.
- the methodology developed relies on the use of biomass from abundant plants. Our methodology is thus a sustainable way of managing the biomass from spreading of *E. crassipes*.

The results obtained here open up new perspectives in the field of phytoremediation and pollution management by natural means.

## Methods

### General

All chemicals were purchased from Alfa Aesar or Sigma-Aldrich.

Gas chromatography-mass spectrometry (GC-MS) analyses were carried out by using a TRACE^TM^ 1300 apparatus equipped with an ISQ^TM^ QD mass spectrometer detector (Thermo Fisher Scientific). The following chromatographic system was used for analytical experiments: GC-MS (Thermo TG-5SilMS, 20 m, 0.18 µm) with hydrogen as carrier gas [80 °C (0.5 min), followed by linear gradient from 80 to 280 °C (5 min)] at a flow rate of 0.8 mL/min.

#### Calibration curve

The identification of the samples was based on comparison of its retention time with those of the standard compound.

A straight line of the calibration was determined with standard samples. The calibration curve was constructed by plotting peak areas of 6 corresponding concentrations 0.045, 0.083, 0.197, 0.354, 0.451, 0.522 μgmol.ml^−1^.

Three injections of each solution were used to obtain the curve. The calibration curve was linear (R^2^ = 0.9969). The limits of detection and quantification were established at 0.028 μgmol.ml^−1^ and 0.045 μgmol.ml^−1^ respectively. The relative standard deviations (RSD) of the results obtained were satisfactory (0.6/0.9/1.1/2.8/8.2/16.8%).

The calibration curve was performed two times and the experiment of quantification was tested four times.

#### Flash chromatography

Flash chromatography was performed on an Interchim Puriflash 430 by using an UV detector.

Silica gel 60 (230–400 µm, Merck) was used for CC. The methanolic extract of root was chrmatographed with cyclohexane/AcOEt gradient. Fraction 1 was eluted with cyclohexane/AcOEt 8.5 :1.5, v/v, fractions 2 and 3 with 5:5, v/v as mobile phase.

#### UHPLC-HRMS analysis

UHPLC-HRMS (SYNAPT G2-S (Waters Corporation, Manchester, UK) equipped with an ESI source was employed.

High resolution electrospray ionization mass spectrometry (HR-ESI-MS) were acquired in positive or negative ion mode. The conditions were the following: capillary voltage 3000 V; cone voltage 20 V, dry gas temperature 140 °C, desolvatation temperature = 450 °C, dry gas flow, 1000 L.h-1 and nitrogen as nebulizer gas, Pressure = 6.5 bars.

Analytic UHPLC was performed using a column Kinetex C18 (100 mm × 2.1 mm, 1.7 µm), at 30 °C.

1 ng/µl Leucine Enkephalin was used as standard for internal calibration. The elution gradient is described in the Table 1.

**Table 1:** Elution conditions

Gradient	%A*	%B**
00.0 min	100	0
10.0 min	0	100
12.0 min	0	100
12.1 min	100	0
15.0 min	100	0

*A: Milli-Q Water + 0.1% formic acid.

**B: Acetonitrile (ULC-MS quality) +0.1% formic acid.

### Rhizolfiltration and biosorption experiments

#### Conditions for *E. crassipes* growth

*E. crassipes* was purchased from a specialised grower (Occitanie, France) over two years (in June 2017 and June 2018). The plants were placed in pool water in greenhouses and in confined conditions. Guano was added as fertilizer. Each *E. crassipes* plant was washed by deionized water before experiments.

### Treatment of *E. crassipes* for metal extraction experiments

#### Rhizolfiltration experiments

Each plant was transferred in a container and was exposed to different synthetic solutions for 6 hours at room temperature. Each synthetic solutions contained 3 μmol of one pollutant. When the pollutants (**1**, **2**) were not miscible with water, the mixture water/isopropanol (1:1, v/v) was used.

**Figure 10:**
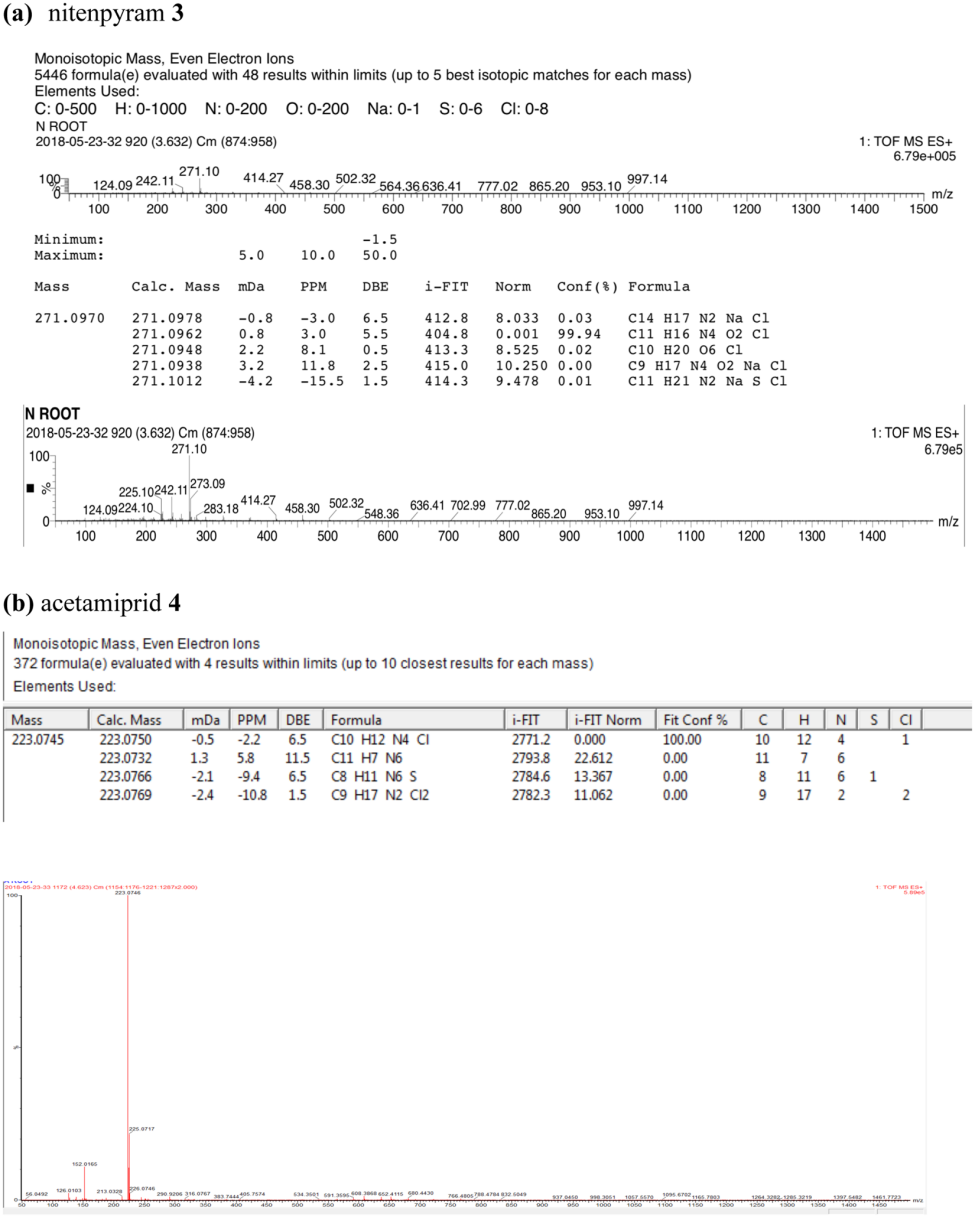
Representative HRMS chromatograms after UHPLC of extracts of *E. crassipes*: (**a**) nitenpyram **3** and (**b**) acetamiprid **4**.

After stirring, roots were harvested, cut, filtered. After extraction by EtOH, they were analyzed by GC-MS (**1**, **2**) or UHPLC-HRMS (**3**, **4**, Fig. 10). The samples were injected, in triplicate, directly into GC-MS or UHPLC-HRMS.

#### Biosorption experiments

Roots of *E. crassipes* were harvested and dried at 80 °C overnight. The dried roots were ground using a vegetal grinder to give the root powder. 50 mg of *E. crassipes* root powder was added to 50 mL of a 10 mg 2:1 solution of *i*-PrOH/H_2_O. After 1 hour of stirring, both the phthalate **1** and pentabromodiphenyl ether **2** extracted from the vegetable powder were identified by GC-MS.

#### FT-IR analysis of the *E. crassipes* roots

IR spectra were recorded on a Perkin-Elmer Spectrum 100 FT-IR® spectrometer in ATR (Attenuated Total Reflexion) mode. The number of scans was 32, the resolution was 1 point per cm^−1^. The acquisition was done from 650 to 4000 cm^−1^. The detector used was a DTGS (Deuterared-TriGlycine Sulfate). The background was done in air.

The calculation of the areas of each peak was done by fitting using a combination of Gaussian and Lorentzian functions, in order to better adjust the experimental peaks. This calculation was done after a baseline correction (polynomial function of order 5) and an extraction of the interest zone in cm^−1^, in order to limit the number of peaks to be fit. This treatment was performed on the Labspec® 4.18 software (Table 2).

**Table 2:** Results of mathematical fitting of ATR-FT-IR.

p	a	w	g	Min	Max	Formula	Area
1469.46	0.00974648	97.2238	0	0	1	GaussLoren()	0.317773
1471.41	0.00025878	94.6679	0.337991	0	1	GaussLoren()	0.00767223
1478.67	0.00598527	99.8057	0.595097	0	1	GaussLoren()	0.194982
1512.72	0.00522003	18.6674	1	0	1	GaussLoren()	0.0537867
1517.51	0.0101003	76.7291	0.0445792	0	1	GaussLoren()	0.452041
1534.84	0.0046722	94.1967	0.018596	0	1	GaussLoren()	0.26171
1549.16	0.00285933	26.8663	1	0	1	GaussLoren()	0.0424025
1560.94	0.00319983	97.4718	0.727989	0	1	GaussLoren()	0.176268
1575.01	0.00567833	98.7934	0.973408	0	1	GaussLoren()	0.308198
1588.39	0.0068697	99.4369	0.999942	0	1	GaussLoren()	0.375569
1594.54	0.0116425	99.7659	0.999967	0	1	GaussLoren()	0.639462
1607.33	0.0230517	59.2155	0.422239	0	1	GaussLoren()	0.886496
1631.03	0.0118723	33.4501	0.47732	0	1	GaussLoren()	0.2631
1650.76	0.0134437	93.1424	1	0	1	GaussLoren()	0.691161
1653.39	0.0144369	50.4987	0.3869	0	1	GaussLoren()	0.48568
1657.67	0.0107601	50.7527	0.474435	0	1	GaussLoren()	0.354741
1693	0.00556285	42.6967	1	0	1	GaussLoren()	0.131103
1720.86	0.00287499	18.8239	1	0	1	GaussLoren()	0.0298721
1736.02	0.00449175	26.4704	1	0	1	GaussLoren()	0.065629

p = Peak Position; a = coefficient; w = width at half height; g = contribution of Gaussian function (between Min = 0 and Max = 1); Formula = Gaussian and Lorentzian functions combinaison; Area = Area of the fit peak.

Baseline correction: Automatic fitting with a polynomial function of order 5.

Extraction Zone: 1500 to 1800 cm^−1^
